# Evaluation of the impact of transient interruption of antiangiogenic treatment using ultrasound-based techniques in a murine model of hepatocellular carcinoma

**DOI:** 10.1186/1471-2407-14-403

**Published:** 2014-06-04

**Authors:** Sara Marinelli, Veronica Salvatore, Marco Baron Toaldo, Maddalena Milazzo, Luca Croci, Laura Venerandi, Anna Pecorelli, Chiara Palamà, Alessia Diana, Luigi Bolondi, Fabio Piscaglia

**Affiliations:** 1 Department of Medical and Surgical Sciences, University of Bologna and S. Orsola-Malpighi Hospital, Bologna, Italy; 2 Department of Veterinary Medical Science, University of Bologna, Bologna, Italy; 3 Centro di Ricerca Biomedica Applicata, University of Bologna and S. Orsola-Malpighi Hospital, Bologna, Italy

**Keywords:** Hepatocellular carcinoma, Antiangiogenic treatment, Molecular contrast-enhanced ultrasonography, Elastosonography

## Abstract

**Background:**

Development of escape pathways from antiangiogenic treatments was reported to be associated with enhanced tumor aggressiveness and rebound effect was suggested after treatment stop. Aim of the study was to evaluate tumor response simulating different conditions of administration of antiangiogenic treatment (transient or definitive treatment stop) in a mouse model of hepatocellular carcinoma.

**Methods:**

Subcutaneous tumors were created by inoculating 5×10^6^ Huh7 cells into the right flank of 14 nude mice. When tumor size reached 5–10 mm, mice were divided in 3 groups: group 1 was treated with placebo, group 2 was treated with sorafenib (62 mg/kg via gavage) but temporarily suspended from day +5 to +9, whereas in group 3 sorafenib was definitively stopped at day +5. At day +13 all mice were sacrificed, collecting masses for Western-Blot analyses. Volume was calculated with B-mode ultrasonography at day 0, +5, +9, +11 and +13. VEGFR2-targeted contrast-enhanced ultrasound using BR55 (Bracco Imaging) was performed at day +5 and +13 and elastonosography (Esaote) at day +9 and +11 to assess tumor stiffness.

**Results:**

Median growth percentage delta at day +13 versus day 0 was 197% (115–329) in group 1, 81% (48–144) in group 2 and 111% (27–167) in group 3. Median growth delta at day +13 with respect to day +5 was 79% (48–127), 37% (−14128) and 81% (15–87) in groups 1, 2 and 3, respectively. Quantification of targeted-CEUS at day +13 showed higher values in group 3 (509 Arbitrary Units AI, range 293–652) than group 1 (275 AI, range 191–494) and group 2 (181 AI, range 63–318) (*p* = 0.033). Western-Blot analysis demonstrated higher VEGFR2 expression in group 3 with respect to group 1 and 2.

**Conclusions:**

A transient interruption of antiangiogenic treatment does not impede restoration of tumor response, while a definitive interruption tends to stimulate a rebound of angiogenesis to higher level than without treatment.

## Background

Antiangiogenic treatments have become the mainstay of oncologic treatments in a variety of cancers [1-3]. Such treatment does not produce complete tumor necrosis, but delay tumor progression and is therefore to be utilized continuatively as a chronic therapy.

However, even in presence of tumor response, unfortunately adverse events may develop requiring transient or permanent drug interruption. At treatment stopping, neoangiogenesis becomes intensively stimulated through the usual pathways previously blocked by the drug and through alternative pathways induced by the drug treatment, through the activation of pre-existing invasion program or cancer cell phenotypic change and selection of clones resistant to hypoxia [4-6].

A 10-fold higher incidence of invasive carcinomas at 1, 2 and 3 weeks after withhold of therapy stop have been reported as well as a rapid volume increase [4,7]. Thus, the maintenance of antiangiogenic treatments even during progression may be justified in order to prevent such rebound effect of tumor neoangiogenesis [8].

Sorafenib is the only drug currently approved for advanced Hepatocellular Carcinoma (HCC) and acts by blocking Vascular Endotelial Growth Factor Receptor 2 (VEGFR2), Platelet Derived Growth Factor Receptor (PDGFR), Raf-1, B-Raf and c-kit among others [9]. Like other antiangiogenic treatments, it aims at blocking neoangiogenesis and/or tumor cell proliferation rather than acting through a direct cytotoxic necrotizing effect, making dimensional criteria poorly performant to evaluate tumor response.

Molecular contrast-enhanced ultrasound (CEUS) involves the use of molecularly-targeted microbubbles (MBs) with potentialities in oncology ranging from cancer detection or characterization to assessment of response to treatment. In order to avoid streptavidin and biotin as linking agents, which are potentially immunogenic, a new conjugation method has been recently reported, where VEGFR2 targeted lipopeptide are directly incorporated into the MB shell [10]. In this way, they can be used in humans and an early study in 12 patients has already been performed [11]. These targeted MBs allow to identify sites of active neoangiogenesis, like those occurring in tumoral tissue, whilst healthy parenchyma present only minimal and non specific MBs binding [12]. Their binding specificity to VEGFR2, attachment to blood vessels and utility in monitoring antiangiogenetic treatment have already been reported [13,14]. Moreover, a direct correlation between quantification of VEGFR2-targeted CEUS and immunohistochemical analysis has been demonstrated also in very small tumors [15].

Elastosonography is an ultrasound based technique able to evaluate the elastic proprieties of a tissue by analyzing the strain in response to a manual compression in a totally non invasive way [16-18]. We have recently demonstrated its utility in the identification of tumor responding to antiangiogenetic treatment, based on the observation that a softening occurs in good responders at 2 days from the beginning of treatment [19].

The present study aims to evaluate the efficacy of sorafenib, an antiangiogenic drug, in a murine model of HCC submitted to different treatment interruption schedules, correlating treatment outcomes with molecular mechanism explored with novel ultrasound based techniques.

## Methods

### Experimental model

The human cell line Huh7 (ATCC cell bank, VA, USA) was maintained and expanded using standard cell culture technique in high glucose Dulbecco’s Modified Eagle Medium supplemented with L-glutamine, 1% ampicillin/amphotericin B and 10% fetal bovine serum (Gibco, Italy). Heterotopic tumors were created by subcutaneous injection into the right flank of 6–8 weeks old female nude CD1 mice (Charles River, Italy) of 5×10^6^ cells suspended in sterile phosphate-buffered saline (Gibco, Italy) for a total volume of 0.2 mL per injection. Mice were maintained with unrestricted regular mouse chow and water in a temperature- and humidity controlled room kept on a 12-hour light/dark circle and specific pathogen-free environment. Twenty animals have been inoculated and masses grew in 16 of them. Mice were randomized in three groups: group 1 was treated with placebo, group 2 was treated with sorafenib (BAY 43–9006; Bayer, Germany) at a dosage of 62 mg/Kg by oral gavage daily until day +5, then with placebo until day +9, when sorafenib was started again (at the same dosage) until day +13, and group 3 was treated with sorafenib (at the same dosage) until day +5 and then with placebo. Sorafenib was formulated as previously described [9]. Growth of established xenograft tumors was monitored at least twice weekly by ultrasound. The experimental protocol was approved by the veterinary university animal welfare committee (Comitato Etico Scientifico per la Sperimentazione Animale, University of Bologna).

### Ultrasound, elastosonography and molecular imaging experiments

**Figure 1:**
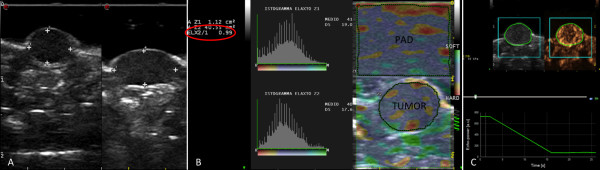
**B-mode, elastonosonography and contrast enhanced ultrasonography with VEGFR2 targeted microbubbles images.** B-mode ultrasonography (panel **A**) was used to calculate tumor dimensions whereas elastosonography (panel **B**) allowed to evaluate the elasticity of the tumor with respect to a pad with costant elasticity. Elasticity ratio is reported (ELX2/1) as well as histogram of elasticity distribution. In panel **C**, contrast enhanced ultrasonography with VEGFR2 targeted microbubbles images immediately before the high mechanical index flash (5 minutes and 59 seconds) is shown (upper part of the panel). Time intensity curve (lower part of panel **C**) was created using a dedicated software to calculate differential targeted enhancement (dTE).

Ultrasound examinations were performed using a MyLab90 Twice (Esaote, Italy) equipped with a broadband 4–13 MHz probe. Mice were anesthetized with an intraperitoneal solution constituted by one part of ketamine 10% (Ketavet, Intervent Production s.r.l., Italy), one part of xylazine 20 mg/mL (Rompun, Bayer AG, Germany) and eight parts of sterile water, for a total of 0.2 mL. Then animals were placed on a temperature controlled heated support to keep constant the body temperature for the whole duration of measurements. A layer of warmed ultrasound gel was placed over the skin of tumors for B-mode, elastosonography and molecular-CEUS examinations (Figure 1). Tumor volume was calculated using the formula: height × width × thickness/2 measured by ultrasound, considering the respective longest diameter (Figure 1). When tumors reached 5–10 mm in diameter, mice were included in the 13-days protocol and then volume was monitored at day 0, +5, +9, +11 and +13.

Elastosonography was performed by a single operator. As explained elsewhere [19], the deformability of tumor tissue in response to a manual strain applied perpendicularly to the skin by the operator is depicted as colour-coded images. The same condition of brightness, contrast, intensity and gain were used in all the examinations as well as attention was paid to scan the tumor across its longest transversal section. A pad of known constant and homogeneous consistency (Zerdine, CIRS, Norfolk Virginia, USA) was interposed between the probe and the tumor because strain imaging modalities do not provide absolute measurements of tissue stiffness. In this way, the elasticity of the tumor was correlated with the same reference standard in all experiments and changes over time in stiffness were assessed by the changes in such ratio. Details of the modality have been described in a previous study of our group [19] and were maintained identical. Three measurements were performed in each tumor during anaesthesia and the mean value was used for following analyses. Higher ratio indicates greater tumor elasticity (i.e. softer tissues) (Figure 1). The entire elastographic procedure lasted approximately 2 minutes per mice and was performed at day +9 and +11 in order to evaluate non invasively the different behaviour of tumours when the treatment with sorafenib was started again in group 2.

For contrast enhanced ultrasonography with VEGFR2-targeted MBs, the probe was placed on a fixed mechanical support in order to maintain the same scanned section of the tumor for the whole duration of the US exam. A contrast specific software (Contrast Tuned Imaging, CnTI) was activated in a dual display modality (B-mode window and contrast window) in order to be sure to scan the correct area. The following US setting were used and maintained for all experiments: dynamic range, 7 dB; acoustic power, 30 kPa; mechanical index, 0.03; depth, 22–37 mm; midscale time-gain compensation, linear.

VEGFR-2 targeted MBs contrast agent (BR55, Bracco Imaging, Switzerland) was reconstituted by injecting 2 ml of a sterile 5% glucose solution through the septum of the vial. A volume of 1.7 μl/g of MB suspension (2.4×10^7^ MBs) was injected into the mouse venous circulation through the retro-orbital sinus. Immediately after the injection a 30 seconds continuous video clip was acquired at low MI. A second 30 seconds long video clip was acquired starting from 5 minutes and 55 seconds after injection. At 6 minutes the MBs present in the field of view containing the tumor were destroyed by temporarily (1 second) increasing the acoustic power (MI 1.9). The subsequent 18 seconds were utilized to assess still circulating MBs. This process is called destruction-replenishment analysis [20]. The same procedure protocol was repeated at day +5 (when group 2 and group 3 were on treatment) and at day +13 (when only group 2 was on treatment).

Post processing analysis of the destruction-replenishment video clips, recorded as DICOM files, was performed using a prototype software (Bracco Suisse SA, Switzerland). This software is designed to quantify contrast echo-power within a region of interest (ROI) enclosing the entire tumor area. Before proceeding to quantification, the analysis first applies linearization of signal intensity at the pixel levels to reverse the effects of “log” compression in the ultrasound system. Contrast enhancement in the ROI was expressed as relative echo-power values, which are proportional to the number of MBs in the selected ROI. The software automatically recognizes the high MI frames, and it considers for quantification only the 2 seconds before the high MI period and the 10 seconds following the 15^th^ second after the flash. The signal intensity after destruction (TE_ad_) was subtracted from signal intensity determined before destruction (TE_bd_) in order to obtain the differential targeted enhancement (dTE = TE_bd_-TE_ad_). Since the TE_bd_ expresses both the circulating and the bound MBs, whereas TE_ad_ only the circulating MBs, the difference between them (dTE) represents a numeric value proportional to the amount of MBs bound to the target receptor VEGFR2 (Figure 1).

The 30 first seconds clip taken in the arterial phase were evaluated blindly and independently by two operators in order to quantify visually the percentage of non enhanced (hence non perfused) areas. Rate of non-enhanced areas were quantified using a 10% step scale through visualization of tumor perfusion at peak enhancement. In case of mismatch, the final decision was achieved by consensus.

### Necropsy

At day +13 after the last measurement and still under anaesthesia, all animals were euthanized by 0.1 mL of a solution of embutramide, mebezonium iodide and tetracaine hydrochloride (Tanax, Intervet Italia s.r.l., Italy) and tumors were cut in two halves: one immersed in liquid nitrogen and then stored at −80°C for Western-Blot analyses and one stored in 4% paraformaldehyde and used for histopathology.

### Western-blot analysis

Two polyclonal antibodies against VEGFR2 (Cell Signaling Technology, Inc. Danversa, MA, USA) (diluted at 1:1000) and HIF-1α (Santa Cruz Biotechnology, Inc. Santa Cruz, CA, USA) (diluted at 1:200) were incubated separately for 16 hours at 4°C. A horseradish conjugated secondary antibody (labeled polymer-HRP antirabbit, Envision system DAKO Cytomation, Carpinteria, CA, USA) was incubated for 45 minutes at room temperature and the corresponding band was revealed using the enhanced chemoluminescence method (Amersham, UK). Digital images of autoradiographies were acquired and quantified with ChemiDoc™ XRS + (Image Lab™ Software, Bio-Rad).

Images were calibrated against a reference autoradiography and given in relative density units (d.u.). After autoradiography acquisition, the membranes were stripped and reprobed for two hours at room temperature with anti- β-actin antibody (Santa Cruz Biotechnology, Inc. Santa Cruz, CA, USA) to normalize protein loading. A ratio between VEGFR2 or HIF-1α and β-actin corresponding bands was used to quantify the levels of each protein (normalized value).

Three randomly selected samples of each group were used for Western-Blot analyses.

### Histopathology

Tumors samples, taken at autopsy and fixed in 10% phosphate-buffered formalin for 12 to 24 hours, were embedded in paraffin for histological processing. Four-micron sections were then stained with standard hematoxylin-eosin for histological examination that was performed by two examiners blinded to treatment protocols, assessing the presence of necrosis and of vascular structures. In case of discrepancy, a consensus was reached after discussion.

### Statistical evaluation

Data are presented as median (range). Differences between groups were assessed using the Mann–Whitney test or Kruskal-Wallis test as appropriate. Differences among different time points in the same group were analyzed using the Wilcoxon signed Rank test. Spearman test was used for correlation analysis. Modifications among different time points of various variables (volume, elasticity and dTE), expressed as percentage delta, were calculated using the formula [(final value − starting value)/starting value] %. P < 0.05 was considered significant. Statistical analysis was performed using SPSS 16.0 (Chicago Il, USA).

## Results

### Tumor size increase

The study group comprised 14 mice, 4 included in group 1, 6 in group 2 and 4 in group 3; in fact 2 animals with fast growing and large masses (1 in group 1 and 1 in group 3) out of the 16 harbouring tumors were found dead in the cage before reassessment and hence excluded from the analysis. Tumor volume at day 0 was 143 mm^3^ (105–408) in group 1, 174 mm^3^ (128–190) in group 2 and 121 mm^3^ (75–648) in group 3 (*p* = n.s.). At day +13, tumor volume was 706 mm^3^ (308–1748) in group 1, 277 mm^3^ (85–465) in group 2 and 443 mm^3^ (187–1118) in group 3, with an increase of 197% (115–329), 81% (48–144) and 111% (27–167), respectively (*p* = n.s.).

**Figure 2:**
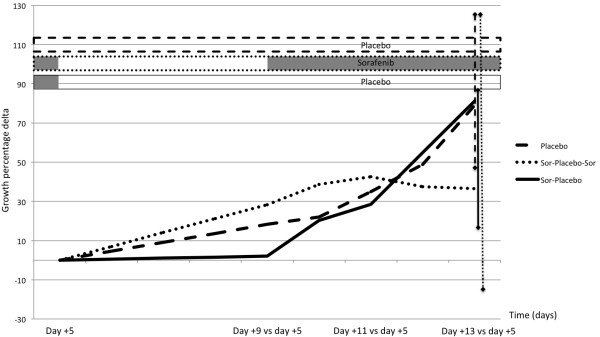
**Growth percentage delta with respect to day +5.** Growth percentage delta with respect to day 5, when treatment was temporarily stopped in group 2 and definitively in group 3. Resuming treatment administration in group 2 at day +9 prevented further increase in tumor dimensions from day +9 to day +13 (+37% day +13 versus day +9, range −14, +127) whereas the definitive treatment stop in group 3 induced a tumor growth comparable to that of group 1 (placebo group) (respectively 81%, range 15–87, and 79%, range 48–127). Median values and range are reported.

When tumor volume at day +13 (end of study) were compared to day +5, when treatment was stopped in group 2 (temporarily) and in group 3 (definitively), the growth increase was 79% (48–127) in group 1, 37% (−14 − +127) in group 2 and 81% (15–87) in group 3 (*p* = n.s.), with a relative increase of 1.8, 1.4 and 1.8 folds (Figure 2).

### Contrast enhanced ultrasonography with VEGFR2-targeted MBs

Contrast-enhanced ultrasonography with VEGFR2-targeted MBs was performed at day +5 (when sorafenib treatment was stopped temporarily in group 2 and definitively in group 3) and at day +13. dTE values at day +5 were 293 a.u. (121–1340) in group 1, 190 a.u. (62–255) in group 2 and 132 a.u. (79–786) in group 3 (*p* = n.s.). dTE values at day +13 were 275 a.u. (191–494) in group 1, 181 (65–318) in group 2 and 509 a.u. (193–652) in group 3 (*p* = 0.033 among three groups and *p* = 0.019 comparing only group 2 and group 3 between them).

dTE percentage delta were +5% (−51 − +91) in group 1, −17% (−46 − +81) in group 2 and +266% (+119 − +730) in group 3 (*p* = 0.018 among three groups, *p* = 0.010 between group 2 and group 3 and *p* = 0.029 between group 1 and 3).

dTE values remained quite constant in group 1 and 2 (median change of 1 and 0.8 fold, respectively) while markedly increased in group 3 (median increase of 3.7 fold), suggesting over expression of VEGFR2 in response to the definitive stop of the treatment, considering both absolute values and relative changes in dTE between G5 and G13 (in group 3 *p* = 0.068).

**Figure 3:**
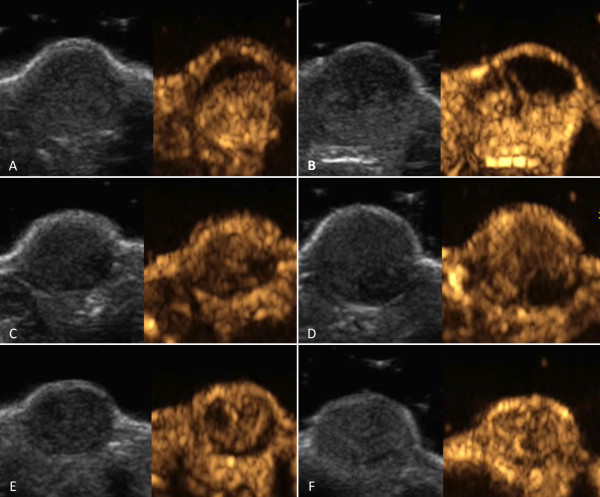
**Tumor perfusion at peak enhancement.** Representative images of contrast enhanced ultrasonography at peak enhancement in group 1 (panels **A** and **B**), group 2 (panels **C** and **D**) and group 3 (panels **E** and **F**) at day + 5 (panels **A**, **C** and **E**) and at day +13 (panels **B**, **D** and **F**). Corresponding B-mode images are shown. At day +13, necrotic areas are present in group 1 (placebo group) and group 2 (sorafenib-placebo-sorafenib treatment) tumors whereas all tumor areas in group 3 tumors (sorafenib treatment stopped at day +5) are perfused, which might be speculated to derive from overstimulation of neoangiogenesis.

We further evaluated the percentage of enhancement in the arterial phase, in order to identify the rate of non perfused (and theoretically hypoxic) areas. Percentage of non enhanced (necrotic) areas is reported in Table 1. In particular, at day +13 it was higher in group 1 (30%, 20–50) than in group 2 (15%, 0–30) but especially than in group 3 (5%, 0–10;), suggesting the effective over stimulated neoangiogenesis able to perfuse quite all tumor areas (Figure 3). The difference among the three groups at day +13 tended to reach the statistical significance (*p* = 0.059) due to the decrease in necrotic areas in group 3 (*p* = n.s. between group 1 and group 2 and between group 2 and group 3; *p* = 0.019 between group 1 and group 3).

**Table 1:** Percentage of non enhanced areas

	**Day +5**		**Day +11**	
	**%**		**%**	
**Group 1 (Placebo)**	20		20	
	0		20	
	30		50	
	10		40	
Median values		15%		30%
**Group 2 (Sor-Placebo-Sor)**	0		0	
	30		30	
	0		10	
	30		20	
	20		40	
	0		0	
Median values		10%		15%
**Group 3 (Sor-Placebo)**	30		10	
	10		0	
	30		10	
	0		0	
Median values		20%		5%
		*p* = n.s.		*p* = 0.059

Rate of non enhanced areas at day +13 was lower in group 3, suggesting that an over stimulated neoangiogenesis is able to perfuse all tumor. Data are expressed as individual values of each mass.

### Western-blot analysis

**Figure 4:**
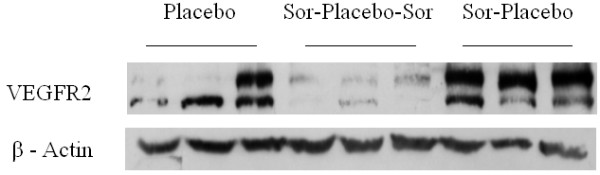
**Western Blot analysis.** Representative images of Western-Blot analysis of VEGFR2 protein expression in tumor samples. VEGFR2 levels were higher in group 3 with respect to the other groups.

VEGR2 levels at day +13 were higher in group 3 with respect to the other groups (Figure 4), consistent with contrast-enhanced ultrasonography with VEGFR2-targeted MBs. In particular, VEGFR2 levels were 0.33 d.u. in group 1, 0.05 d.u. in group 2 and 2.01 d.u. in group 3 (*p* = 0.061 among the three groups; *p* = 0.05 between group 1 and group 3 and between group 2 and group 3; *p* = n.s. between group 1 and group 2).

Confirmation of the analysis of the arterial enhancement showing necrotic percentage observed with molecular CEUS emerged from HIF-1α analysis. Indeed, slightly higher levels of this protein were present in group 1 (0.52 d.u.) with respect to group 2 (0.47 d.u.) but especially to group 3 (0.30 d.u.) (*p* = n.s.), supporting the idea that the over-stimulated neoangiogenesis in group 3 is able to perfuse quite all tumor areas, reducing hypoxic regions. In order to exclude any influence of tumor dimension on HIF-1α expression, a correlation between these two parameters was performed without reaching any statistical significance (*p* = n.s.).

### Histopathology

**Figure 5:**
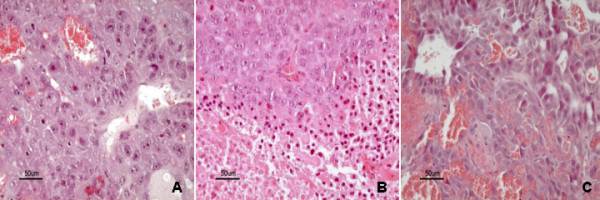
**Histopathology.** Representative pictures of hematoxylin-eosin stained samples from group 1 (placebo, panel **A**), group 2 (Sor-Placebo-Sor, panel **B**) and from group 3 (Sor-Placebo, panel **C**). Necrotic areas were present in all tumor samples from the three groups. However extensive necrotic areas, either solid or colliquative, were mostly evident in group 2.

Histopathological analysis of the 14 tumor samples showed a heterogeneous pattern, with well represented stromal tissue supporting the xenograft growth and large neovascular structures in their context. Tumors specimens of group 1 and group 3 appeared richer in vessels as compared to group 2. In particular, many neoformed vessels as well as lakes of extravasated erythrocytes were seen in groups 1 and 3 (Figure 5, panels A and C), and were almost absent in group 2 (Figure 5, panel B). Necrotic areas, characterized by solid or colliquative changes with dissolution of cell membranes and faint or absent nuclei, were seen in all specimens from the three groups. However they were more prominent in tumors samples from group 2, in which it was often possible to additionally observe picnotic and fragmented nuclei, suggesting apoptotic changes. Remarkably, nuclei were morphologically different in the three groups. Namely in group 1 and group 3 tumor nuclei appeared vesicular, a typical appearance of cells often associated with secretion processes.

### Changes in elasticity using elastosonography

Elastosonography measurements were performed at day +9 and +11 in order to evaluate the response of tumors to the re-introduction of sorafenib treatment in group 2, with respect to the other groups treated with placebo.

**Figure 6:**
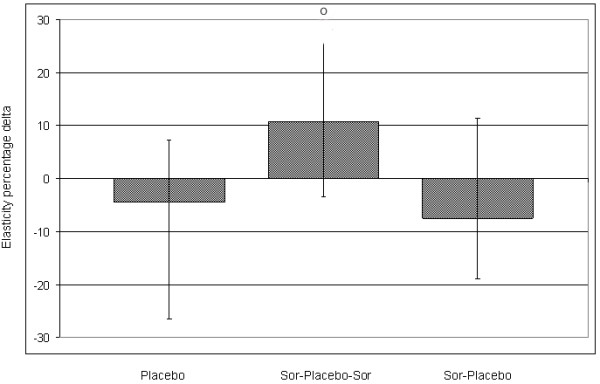
**Elasticity percentage delta.** Elasticity values increased (which corresponds to a tissue softening) only in group 2 when treatment was restarted (+10.8%, range −3.5, +55). Conversely, treatment elasticity tended to decrease under placebo (−4.6%, range −26.7 − +7.3 in group 1 and −7.5%, range −19.02, +11.3 in group 3). Median values and range are reported.

Elasticity ratio at day +9 was 1.34 (0.87–1.49) in group 1, 1.10 (1.04-1.45) in group 2 and 1.14 (1.09-1.19) in group 3 (*p* = n.s.). At day +11 elasticity was 1.15 (0.93-1.42) in group 1, 1.33 (1.07-1.65) in group 2 and 1.08 (0.88-1.28) in group 3 (*p* = n.s.). Elasticity increased (corresponding to tissue softening) only in group 2, confirming our previous results that an increase in elasticity is an early indicator of tumor response [19]. In particular, elasticity percentage delta were −4.56% (−26.65 − +7.28) in group 1, +10.79% (−3.45 − +55%) in group 2 and −7.50% (−19.02 − +11.27) in group 3 (Figure 6) (*p* = n.s.).

## Discussion

This study evaluated the effect of transient sorafenib halting in HCC using VEGFR2-targeted MBs and elastosonography. We demonstrated that an early and short interruption of antioangiogenic treatment do not avoid restoration of tumor response while a definitive interruption stimulates angiogenesis to higher levels than even in absence of any treatment.

In animal studies a vascular regrowth after angioangiogenic therapy interruption has been reported to be already present at 2 days after withdrawal [7,21] as well an enhanced distant metastatization [22]. The proposed mechanisms that can play a role in these settings are an upregulation of proangiogenic cytokines and growth factors, the mobilization of bone-marrow derived cells, but also host micro-environmental response to multitarget drugs [22]. The rebound progression is primarily evident at a vascular level [23] and tumors are completely vascularized 7 days after treatment withdrawal [21]. Beside animal models, this phenomenon has been suggested also in few human patients with brain or renal cancers, where the discontinuation of treatment led to higher risk of progression and metastatization [23,24].

In the present study we demonstrated the rebound progression using imaging methods, namely molecular CEUS and elastosonography. Indeed, the higher expression of VEGFR2 demonstrated by molecular CEUS represents the stimulated neoangiogenesis that occurs after sorafenib withdrawal. On the contrary, the further response of tumor submitted to a second round of treatment is mirrored by the downregulation in VEGFR2, seen as well with VEGFR2-targeted MBs. The confirmation of this further response, beside dimensional decrease, derives from elastosonography results, where a softening of treated tumors occurred.

HIF-1α represents a key factor in tumor angiogenesis, being able to activate the transcription of VEGF. During hypoxia, the activity of hydroxylase is inhibited by the low oxygen concentration, stabilizing HIF-1α, which is thus able to translocate into the nucleus where dimerizes with HIF-1β to activate transcriptional target genes. Our results are in keeping with others showing that sorafenib inhibits the synthesis of HIF-1α, leading to a decreased expression of VEGF [25]. On the other hand, a rapid growth itself is able to induce hypoxia, and thus the higher expression of HIF-1α in group 1 is justified. Indeed, the release of other proangiogenic factors beyond VEGF like placenta growth factor (PIGF), fibroblast growth factor and others can be stimulated to supply the hypoxic growing tumor [26]. Finally and more interestingly, the rebound neoangiogenesis that occurs in case of sorafenib definitive withdrawal allows a quite complete tumor perfusion (as demonstrated also by the arterial enhancement quantification) leaving only minimal hypoxic areas and thus leading to a lower expression of HIF-1α (as demonstrated in our study).

The consequence of these observations for the clinical practice is the awareness of rebound neoangiogenesis in case of definite drug withdrawal. It could be speculated therefore a benefit of treatment maintenance where other therapeutical options are not available and the patient would be attended only with best supportive care. Moreover, in case of occurrence of adverse events, if not severe, a dosage reduction may be recommended instead of temporary interruption. Worth to remind that, the protocol of the sorafenib registration trial [1] which showed a survival benefit did not include to stop treatment at the moment of documentation of radiologic progression but only when additionally also symptomatic progression had taken place, so that patients were kept under antiangiogenic therapy for a longer time, possibly preventing the negative effects of a rebound action.

The following limitations of the study need to be mentioned. Whilst a complete revascularization has been reported to be present already at 1 week after drug interruption, the steady state of drug concentration is reached within 7 days and the half-life of sorafenib is 25–48 hours, thus the timing of interruption, re-introduction and final evaluation may be suboptimal [21]. Nevertheless, we suppose that a long-lasting treatment would lead to more pronounced neoangiogenic rebound, but this hypothesis has to be tested in the future. Moreover, it would be of interest to test tumor response following different length of treatment interruption, as different interruptions take place in the clinical practice in case of recurring adverse events. Limitations related to the model are intrinsic in any preclinical experiment and our results would require validation in the human clinical setting, which however cannot be tested in a trial.

## Conclusions

In conclusion, the present study supports the concept of a neoangiogenetic rebound after sorafenib treatment withdrawal in a murine model of HCC. Moreover, the identification of over-expression of VEGFR2 through molecular-CEUS, a well-established new technique for imaging neoangiogenis in small animals, suggests it as a potential tool for human assessment in the future.

## Abbreviations

HCC: Hepatocellular carcinoma; VEGFR2: Vascular endotelial growth factor receptor 2; PDGFR: Platelet derived growth factor receptor; CEUS: Contrast-enhanced ultrasonography; MB: Microbubble; ROI: Region of interest; MI: Mechanical index; TE: Targeted enhancement; dTE: Differential targeted enhancement; HIF: Hypoxia inducible factor.

## Competing interests

Prof Luigi Bolondi: Bayer AG (speaker fee, advisory board), Bristol-Myers Squibb (research grant, advisory board), Bracco (research grant), Roche (speaker fee). Dr. Fabio Piscaglia: Bayer AG (speaker fee, advisory board), Bracco (speaker fee), Siemens Healthcare (speaker fee), Roche (speaker fee). The other authors declare that they have no competing interests.

## Authors’ contributions

SM performed the animal experiments, including model creation and gave important contribution to manuscript preparation; VS acquired funding, conceived and designed the protocol, participated to the experiments, interpreted data, performed statistical analysis and wrote the manuscript; MBT have made substantial contribution to study design, performed the experiments and critically revised the manuscript for important intellectual content; MM performed cell culture, western blot analysis, animal care and has taken part to imaging procedures; LC, LV, AP and CP performed imaging procedures, analysed and interpreted data; AD gave substantial contribution to conception and study design and in data interpretation; LB acquired funding and gave substantial contribution to conception and study design and in data interpretation; FP conceived the protocol, interpreted data and has been substantially involved in manuscript preparation. All authors read and approved the final manuscript.

## Pre-publication history

The pre-publication history for this paper can be accessed here:

http://www.biomedcentral.com/1471-2407/14/403/prepub
